# Resposta à Carta às Editoras de Domingues et al.

**DOI:** 10.1590/0102-311XPT165123

**Published:** 2023-11-13

**Authors:** Michelle Elaine Siqueira Ferreira, Raquel Zanatta Coutinho, Bernardo Lanza Queiroz

**Affiliations:** 1 Universidade Federal de Minas Gerais, Belo Horizonte, Brasil.

A urgência de um sistema de vigilância de *near miss* materno, complementar ao sistema de vigilância de óbito materno, no Brasil, emerge da necessidade de compreensão sobre os fatores associados à estagnação do declínio da razão de mortalidade materna (RMM) - mantida em cerca de 60 óbitos por 100 mil nascidos vivos desde meados de 2015, valor que atingiu 110 óbitos maternos por 100 mil nascidos vivos em 2021 ^1^^,^^2^ - e de avaliação das políticas voltadas à saúde materna e infantil, para que sejam adaptadas continuamente.

Embora, no espectro da morbimortalidade materna, os desfechos maternos graves representem uma pequena parcela das condições que gestantes e puérperas podem experimentar durante o ciclo gravídico-puerperal, a maioria desses desfechos está associada a causas evitáveis ^3^. Nesse contexto, ainda que a vigilância do óbito materno tenha contribuído para a elucidação das suas principais causas, os entraves que ainda mantêm a RMM acima do pactuado pelo país carecem de novas evidências. Assim, tendo em vista que os casos de *near miss* materno são mais prevalentes que os de óbito e considerando a similaridade entre os dois desfechos no que tange às principais causas ^4^^,^^5^, o *near miss* materno é um melhor indicador dos avanços e das falhas na assistência obstétrica do que as demais morbidades maternas, que não necessariamente levam gestantes ou puérperas a uma disfunção orgânica e, consequentemente, ao óbito caso não recebam os cuidados necessários.

Pensando na potencialidade da investigação dos eventos de *near miss* materno, em complementariedade à investigação dos óbitos maternos, regulamentada no ano 2008, Ferreira et al. ^6^, no ensaio intitulado *Morbimortalidade Materna no Brasil e a Urgência de um Sistema Nacional de Vigilância do* Near Miss *Materno*, propuseram a implementação de um sistema de vigilância de *near miss* materno no país. Em resposta ao ensaio, Domingues et al. ^7^ publicaram carta às editoras na qual mencionam a complexidade da implementação do referido sistema de vigilância, ratificando vários argumentos expostos no ensaio original ^6^. Entre os argumentos apresentados, foram pontuados os desafios relacionados à ausência de codificação dos critérios diagnósticos de *near miss* materno na Classificação Internacional de Doenças (CID) e a baixa sensibilidade e o baixo valor preditivo positivo do Sistema de Informação Hospitalar do Sistema Único de Saúde (SIH/SUS) para identificação dos casos ^7^^,^^8^. Além disso, os autores destacam a potencial complexidade que a notificação dos casos via Sistema de Informação de Agravos de Notificação (SINAN) teria caso esse recurso fosse utilizado como instrumento de registro. Em adição, contribuindo para o debate, eles apresentam o Painel de Vigilância de Saúde Materna como uma possibilidade de acompanhamento dos indicadores e sugerem a criação de uma Autorização de Internação Hospitalar (AIH) obstétrica e um Sistema de Informação Perinatal (SIP) ^7^.

Conforme salientado no ensaio, reconhecendo a complexidade de implementação de um novo sistema de vigilância voltado à saúde materna e tendo em vista que a vigilância do óbito é recente, os autores explicitam que o planejamento e a execução de um sistema de vigilância de *near miss* materno deverão ser realizados por equipe multidisciplinar de gestores, pesquisadores, profissionais de saúde e sociedade civil ^6^. Por esse motivo, ainda que de forma muito breve, o SIH/SUS e o SINAN foram sugeridos como possíveis fontes de dados, uma vez que a vigilância em saúde pode e deve ser feita com base em diversas fontes de informação. O objetivo do ensaio foi fomentar a discussão sobre a criação e implementação de um sistema de morbimortalidade materna, também considerando a experiência internacional, que proporcione evidências robustas para o enfrentamento de um problema que o país, de forma mais severa em algumas regiões, não consegue resolver.

No que tange às duas sugestões de registro de dados fornecidas na carta às editoras, não obstante a ausência de classificação do *near miss* materno na CID constituir um entrave para a utilização dos dados do SIH/SUS - tendo em vista os constantes investimentos na melhoria do preenchimento das AIH e o aumento da consistência ^9^, principalmente das variáveis: especialidade do leito, diagnóstico principal e procedimentos realizados -, a criação de uma AIH obstétrica, poderia, caso contenha as variáveis capazes de identificar os casos de near miss materno, contribuir para a recuperação dos eventos diretamente do SIH/SUS.

Quanto à potencialidade do SINAN, a partir da padronização de uma ficha de notificação, similar à do óbito materno, durante a internação ou na consulta pós-alta hospitalar, o profissional de saúde, ao observar a presença de quaisquer dos critérios, poderia proceder à notificação, uma vez que, para caracterização do *near miss* materno, basta que um dos 25 critérios diagnósticos esteja presente ^3^, assim como efetivado no Estado do Paraná ^10^. Ainda que pareça de difícil implementação, não demandaria um esforço maior que aquele expendido na notificação de outros agravos de notificação compulsória relacionada à gestação e ao puerpério (como a sífilis, o HIV, a toxoplasmose), sem mencionar a possibilidade de investigar seus desdobramentos durante a anamnese. Além disso, destaca-se a importância de a vigilância abarcar o contexto da saúde suplementar e privada no Brasil, o que não poderia ser feito somente com a AIH.

Por fim, Domingues et al. ^7^ defendem a implementação de um sistema de vigilância de morbidade materna grave, e não apenas de *near miss* materno, e pontuam que o país já conta com o Painel de Vigilância de Saúde Materna, que consolida informações sobre condições potencialmente ameaçadoras à vida (CPAV). O painel é, de fato, um importante instrumento para gestores e interessados, pois exibe diversos indicadores de saúde materna em âmbito municipal. No entanto, ele não possibilita a análise dos fatores que diferem uma mulher que sobrevive a uma condição materna extremamente grave daquela que vai a óbito, objetivo precípuo da proposta para vigilância do *near miss* materno. Em países avançados na transição obstétrica, onde o número de óbitos maternos é baixo e relacionado a causas inevitáveis ^11^, a vigilância da morbidade materna grave é importante para garantia de um ciclo gravídico-puerperal cada vez mais seguro.

Nos moldes propostos ^5^, cuja sugestão é seguir os padrões da investigação dos óbitos, a vigilância do *near miss* materno se mostra mais eficiente, dado o seu compartilhamento de fatores associados ao óbito e a possibilidade de aprofundamento sobre semelhanças e diferenças nos entraves ao acesso e manejo das complicações. Além disso, é importante destacar o papel da mulher sobrevivente na reflexão crítica sobre as eventuais causas do agravamento do seu quadro de saúde, bem como o que foi crucial para sua sobrevivência.

O ensaio e a carta às editoras ratificam a necessidade de uma equipe multidisciplinar unir esforços e *expertises* para o desenvolvimento de protocolos que abarquem a complexidade do país, a fim de apontar novos caminhos para o alcance de metas antigas ainda não atingidas.
